# Temporal variation in nutritional status and preoperative anemia among patients with retroperitoneal soft tissue sarcoma: a retrospective longitudinal cohort study

**DOI:** 10.1007/s00423-024-03585-5

**Published:** 2025-01-22

**Authors:** Franziska Willis, Anna-Marlen Trunk, Julian Musa, Jonathan M. Harnoss, Moritz J. Strowitzki, Cosima Engerer, Julian-C. Harnoss, Mohammed Al-Saeedi, Markus W. Büchler, Martin Schneider

**Affiliations:** 1 Department of General, Visceral and Transplantation Surgery, University Hospital Heidelberg, Heidelberg, Germany; 2 Division of Translational Pediatric Sarcoma Research (B410), German Cancer Research Center (DKFZ), Heidelberg, Germany; 3 Present Address: Department of General, Visceral, Thoracic and Transplantation Surgery, University of Giessen, Giessen, Germany

**Keywords:** Retroperitoneal soft tissue sarcoma, Multivisceral resection, Overall survival, Nutritional status, Hemoglobin, Preoperative anemia

## Abstract

**Purpose:**

Optimal management of retroperitoneal soft tissue sarcoma (RPS) often requires extensive tumor resections, frequently involving gastrointestinal organs. The impact of these procedures on the nutritional status and hemoglobin (Hb) levels of RPS patients remain unexplored. In this study, we aimed to evaluate preoperative nutritional status as well as the prevalence of anemia in RPS patients, and to investigate longitudinal changes throughout the disease course in order to identify potential strategies for prehabilitation.

**Materials and methods:**

Patients undergoing resection of primary and recurrent RPS at Heidelberg University Hospital were retrospectively analyzed. Changes in nutritional parameters and Hb levels throughout the disease course were analyzed using hierarchical linear regression models. Multivariable Cox regression analyses were performed to identify independent predictors of overall survival. Subgroup analyses were conducted for primary tumors, first, second and third recurrences.

**Results:**

Amongst 370 patients analyzed, comprising 219 with primary disease, we observed neither a significant prevalence of preoperative malnutrition nor notable changes in BMI or serum albumin levels throughout the disease course. Preoperative anemia affected up to 40% of RPS patients, and Hb levels significantly decreased over the course of the disease (*p* = 0.022), particularly in correlation with the number of tumor resections performed (*p* = 0.010). Low preoperative Hb levels were associated with increased 30-day mortality and they were identified as an independent prognostic factor for shorter overall survival in primary RPS as well as in second and third recurrences.

**Conclusion:**

Anemia screening should be performed preoperatively and during regular follow-ups to enable early-on therapy, thus potentially improving patient outcomes in RPS.

**Supplementary Information:**

The online version contains supplementary material available at 10.1007/s00423-024-03585-5.

## Introduction

Retroperitoneal soft tissue sarcomas (RPS) represent a rare and diverse subset of tumors, comprising approximately 1% of adult malignancies [1]. While average 5-year overall survival rates of 67% and 10-year overall survival rates of 46% can be achieved, high recurrence rates (ranging from 40 to 60%) beyond the five-year mark present an ongoing challenge [2–4]. Optimal management revolves around complete tumor resection, the cornerstone in treating both primary and recurrent tumors [5–7]. For liposarcoma, the most frequent histological subtype among RPS, compartmental resection has emerged as the standard surgical approach in an effort to minimize the likelihood of incomplete resection margins [8–10]. This approach entails the comprehensive removal of all adjacent organs and structures associated with the tumor, often requiring extensive multivisceral resections (MVR) [8–10]. MVR is likewise frequently performed in the treatment of other RPS entities [10, 11]. Consequently, patients with RPS undergo extensive surgical procedures, often requiring (partial) resection of organs of the digestive system [11, 12]. The impact of these operations on the nutritional status of RPS patients has not been investigated yet. Only few studies with a limited number of patients have investigated the role of preoperative nutritional status on postoperative outcomes in RPS, revealing high malnutrition rates (40–50%) [13–15]. However, changes of nutritional status due to disease recurrence and repeated operations remain unexplored.

The aim of this study was to assess the preoperative nutritional and anemia status in RPS patients, and to investigate alterations occurring throughout the course of the disease in order to delineate potential prehabilitation strategies.

## Patients and methods

### Patients

Following approval by the ethics committee of the University of Heidelberg (Approval No. S-649/2012), we conducted a retrospective analysis encompassing all patients who underwent surgical resection for primary, recurrent, or metastatic RPS between October 2001 and December 2019 at Heidelberg University Hospital, Department of General, Visceral, and Transplantation Surgery (tertiary care center). Patients with gastrointestinal stromal tumors and those with embryonal, pediatric, or gynecological soft tissue sarcomas were excluded from the study.

In all patients, the primary objective was to achieve macroscopically complete tumor resection, including en-bloc resection of adjacent organs (comprehensive resection) in case of liposarcoma histology [8–10]. All other entities were treated to achieve microscopically complete tumor resection margins. Decisions regarding the necessity of re-resection for recurrent tumors as well as the utilization of additional chemotherapy and radiation therapy were made on a case-by-case basis following review by a multidisciplinary tumor board. Postoperative complications were classified according to the Clavien-Dindo classification system and the Comprehensive Complication Index (CCI) [16]. To provide a comprehensive representation of the cumulative burden of postoperative complications over the entire course of the disease, CCI scores from each hospitalization were aggregated resulting in a cumulative CCI score with a minimum value of 0 and no predefined maximum threshold. To evaluate the preoperative nutritional status, preoperative Body Mass Index (BMI), preoperative albumin as surrogate parameter for protein status, and hemoglobin (Hb) as well as the mean corpuscular volume (MCV) and the mean corpuscular hemoglobin (MCH), were assessed. In case of multiple tumor resections at our department, the parameters mentioned above were assessed within two days before each operation. Hypoalbuminemia was defined as serum albumin < 30 g/l. Anemia was classified according to the WHO as Hb concentration of < 12.0 g/dl in women and < 13.0 g/dl in men. Anemia was categorized into mild (Hb 11.0–11.9 g/dl for women and Hb 11.0–12.9 g/dl for men), moderate (Hb 8.0–10.9 g/dl), and severe (Hb < 8.0 g/dl).

The study was registered at the German Clinical Trials Register (DRKS00034988).

### Statistics

Hierarchical linear models (HLM) were performed to evaluate changes of nutritional parameters (hemoglobin, albumin, BMI), which were assessed repeatedly during the course of the disease. For this purpose, for each patient with more than one tumor resection performed at our department, preoperative parameters before the initial and before the last surgery were taken into account. In addition, we investigated whether changes in these parameters were associated with the performance of MVR (defined as resection of three or more organs), the number of surgeries and the cumulative complication burden during the entire course of the disease. This was realized by introducing 2-way interaction terms. The estimations in the linear hierarchical models were computed as maximum likelihood estimators using the MIXED procedure in SPSS.

Multivariable linear regression models were used to test associations between nutritional parameters and the CCI as well as the length of hospital stay (LoS). To correct for non-normal/heteroskedastic residuals, wild bootstrapping with 2000 replications was performed [17]. Regression models were only calculated when the ANOVA indicated a good fit for the data (*p* ≤ 0.05). In case of dichotomous dependent variables (transfusion of blood components) a logistic regression analysis was performed.

Overall survival (OS) was defined as the date of surgery to the date of death. For alive patients, follow-up was censored at the date of last disease assessment.

Survival was estimated applying the Kaplan-Meier method and is presented as median with 95% confidence interval (CI). Multivariable Cox regression analyses including all potential predictors derived from the univariate Kaplan-Meier analysis (log rank: *p* < 0.1) were performed to identify independent prognostic factors for OS. Possible predictors were resection status (R0/R1 vs. R2), grading according to the Fédération Nationale des Centres de Lutte Contre le Cancer (FNCLCC), histologic subtype, tumor size, MVR, multifocality, patient age, and sex. For nutritional parameters only albumin and Hb were considered to avoid bias due to too many missing values regarding BMI.

Resection status, tumor grade, histology, MVR, multifocality and sex were modeled as categorical using dummy variables. Age, tumor size, albumin and Hb were modeled as continuous variables. In respect of number of events, we used forward and backwards step-wise variable selection [18]. 

**Fig. 1:**
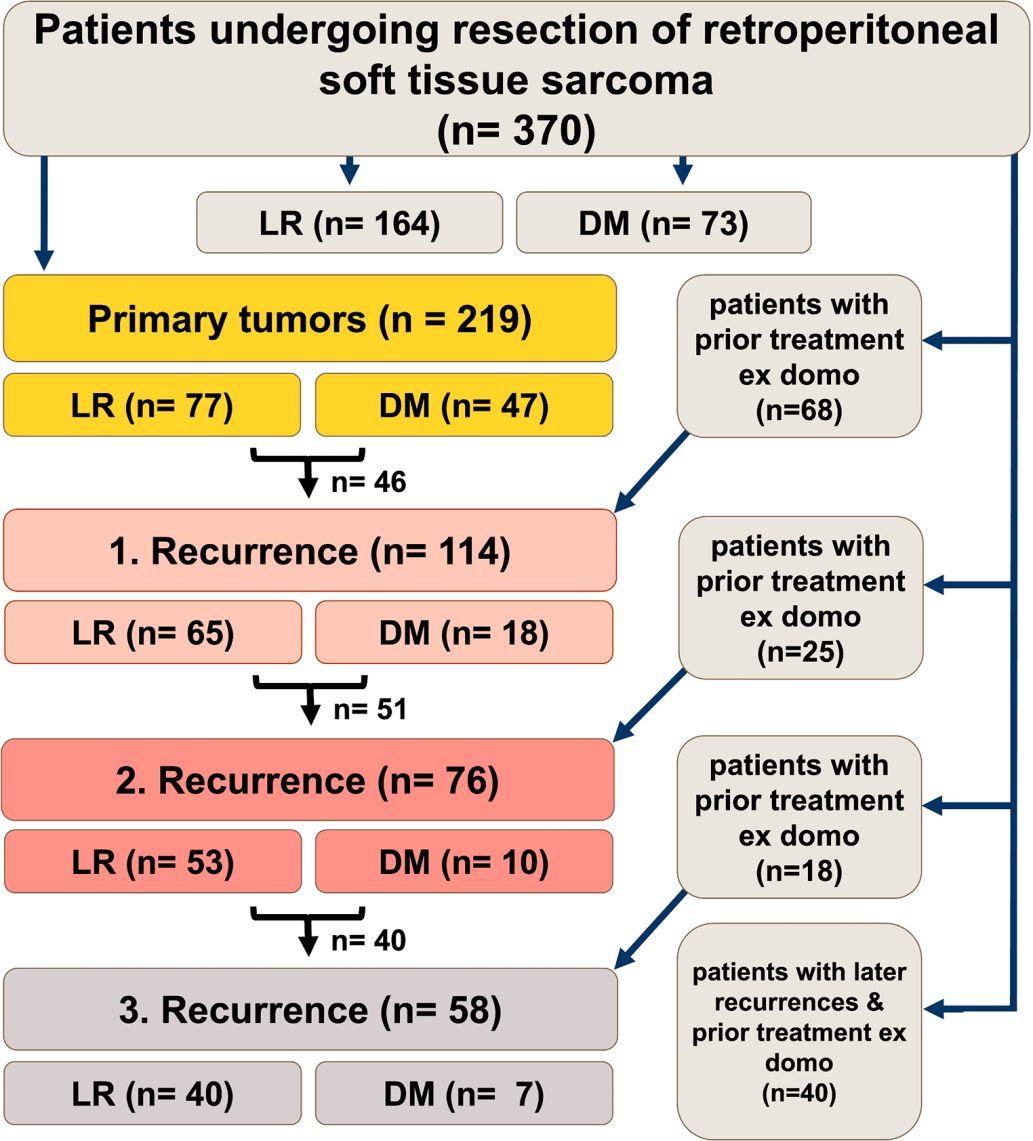
Overview of patients included in this analysis. In total, 370 patients undergoing resection of retroperitoneal soft tissue sarcoma (RPS) were included. 164 patients developed local recurrence (LR), and 73 patients developed distant metastases (DM). 219 patients presented with primary disease. 151 patients underwent initial treatment of primary disease at other hospitals

Analyses were performed considering patients with primary tumors, first, second and third recurrences (see Fig. 1). Patients with incomplete datasets were excluded.

SPSS (Version 29.0.2.0) was used for all statistical analyses. The level of statistical significance was set to *p* ≤ 0.05 (two-tailed).

## Results

### Clinicopathological features and nutritional status

A total of 370 patients who underwent resection of RPS at the University of Heidelberg Department of General, Visceral, and Transplantation Surgery between October 2001 and December 2019 were identified. The analysis included 219 patients with primary disease, 114 patients with first recurrences, 76 patients with second recurrences, and 58 patients with third recurrences (Fig. 1). Demographic, clinicopathological, and treatment details are presented in Table 1.

**Table 1:** Demographic, clinical, and pathological characteristics

	ALL PATIENTS			PRIMARY TUMORS			1st recurrence			2nd recurrence			3rd Recurrence		
n	370			219			114			76			58		
**AGE**. median (IQR). years	58.5	(47.0–66.0)		57.0	(49.0–67.0)		59.0	(50.0-67.3)		60.0	(53.4–68.0)		61.5	(53.0–68.0)	
**SEX** (%)															
Male	195	(52.7)		114	(52.1)		63	(55.3)		46	(60.5)		29	(50.0)	
Female	175	(47.3)		105	(47.9)		51	(44.7)		30	(39.5)		29	(50.0)	
**TUMOR PRESENTATION** (%)															
Primary tumors	219	(59.2)		219	(100.0)		0	(0.0)		0	(0.0)		0	(0.0)	
Local recurrence	122	(33.0)		0	(0.0)		109	(95.6)		70	(92.1)		54	(93.1)	
Distant metastases	11	(3.0)		0	(0.0)		3	(2.6)		4	(5.3)		1	(1.7)	
Primary tumor and distant metastases	8	(2.2)		0	(0.0)		0	(0.0)		0	(0.0)		0	(0.0)	
Local recurrence and distant metastases	10	(2.7)		0	(0.0)		2	(1.8)		2	(2.6)		3	(5.2)	
**HISTOLOGY** (%)															
DD LPS	178	(48.1)		89	(40.6)		72	(63.2)		58	(76.3)		40	(69.0)	
WD LPS	49	(13.2)		33	(15.1)		12	(10.5)		5	(6.6)		11	(19.0)	
LMS	65	(17.6)		46	(21.0)		8	(7.0)		3	(3.9)		3	(5.2)	
NOS	22	(5.9)		14	(6.4)		7	(6.1)		3	(3.9)		0	(0.0)	
Other	39	(10.5)		25	(11.4)		11	(9.6)		4	(5.3)		1	(1.7)	
SFT	17	(4.6)		12	(5.5)		4	(3.5)		3	(3.9)		3	(5.2)	
**TUMOR GRADE** (%)															
G1	47	(12.7)		34	(15.5)										
G2	107	(28.9)		75	(34.2)										
G3	103	(27.8)		61	(27.9)										
does not apply	113	(30.5)		49	(22.4)		114	(100.0)		76	(100.0)		58	(100.0)	
**RESECTION STATUS** (%)															
R0/R1	327	(88.4)		206	(94.1)		106	(93.0)		63	(82.9)		53	(91.4)	
R2	43	(11.6)		13	(5.9)		8	(7.0)		13	(17.1)		5	(8.6)	
**TUMORSIZE**. median (IQR). cm	12.0	(7.0–20.0)		14.0	(8.5–24.0)		7.000	(4.2–12.0)		7.6	(4.8–12.0)		7.0	(3.5–15.6)	
**MULTIFOCALITY** (%)	55		(14.9)	11		(5.0)	22		(19.3)	24		(31.6)	20		(34.5)
**MVR** (%)	153	(41.4)		109	(49.8)		29	(25.4)		14	(18.4)		10	(17.2)	
**BLOOD LOSS**. median (IQR). ml	600		(300–1500)	700		(300–1500)	500		(200–1200)	450		(200–900)	300		(100–625)
**TRANSFUSION** (%)	127	(34.3)		78	(35.6)		31	(27.2)		14	(18.4)		9	(15.5)	
**CLAVIEN DINDO** ≥ 3 (%)	29	(23.2)		50	(22.8)		29	(25.4)		16	(21.1)		9	(15.5)	
CCI. median (%) only w/ complications	33.7	(20.9–51.9)		29.6	(20.9–42.6)		33.5	(20.9–54.2)		33.7	(22.2–47.7)		37.1	(20.9–58.1)	
**30-DAY-MORTALITY** (%)	14	(3.8)		9	(4.1)		1	(0.9)		4	(5.3)		1	(1.7)	
**FOLLOW-UP**. median (IQR). months															
From OP	44.1	(16.9–89.2)		49.3	(19.0-98.8)		42.7	(16.9–85.4)		38.6	(13.4–71.1)		33.7	(17.4–75.5)	
From first diagnosis	65.8	(27.6-128.6)		50.9	(21.9–102.0)		79.6	(40.2-147.7)		92.2	(60.2-160.4)		144.4	(80.7-199.7)	
From OP if alive	73.3	(38.4-118.9)		80.9	(45.1–125.0)		56.8	(30.6-117.7)		42.7	(18.2–78.5)		43.0	(13.9–92.1)	
From first diagnosis if alive	95.3	(50.2-146.4)		84.0	(49.3-131.3)		111.0	(68-153.7)		138.5	(73.5-170.7)		149.7	(93-200.1)	

*IQR* interquartile range, *DD-LPS* dedifferentiated liposarcoma, *WD-LPS* well differentiated liposarcoma, *LMS* leiomyosarcoma, *NOS* undifferentiated sarcoma (not otherwise specified), *SFT* solitary fibrous tumor & other tumors with intermediate malignancy, *MVR* multivisceral resection (≥ 3 organs), *CCI* Comprehensive Complication Index

Details on nutritional status are provided in Table 2. Due to missing information regarding height and weight, BMI values were available for only 250 patients (69%). Among them, the majority was overweight or obese (137 patients, 55% of patients with available datasets), while underweight patients constituted a minority (13 patients, 5% of patients with available datasets). Similar distributions were observed for patients before resection of a primary tumor. However, with an increasing number of resections, the proportion of normal-weight and underweight patients showed an upward trend.

**Table 2:** Overview of parameters assessed for evaluation of nutritional status

*n*	ALL PATIENTS		PRIMARY TUMORS		1st recurrence		2nd recurrence		3rd Recurrence	
	370		219		114		76		58	
**HEIGHT**, median (IQR), cm	172.00	(164.0-180.0)	172.0	(165.0-180.0)	172.0	(164.0-180.0)	174.0	(165.0-183.0)	170.0	(164.0-181.0)
Missing values	113	(30.5)	62	(28.3)	37	(32.5)	14	(18.4)	5	(8.6)
**WEIGHT**, median (IQR), kg	76.0	(65.0–87.0)	76.0	(65.0-87.8)	74.5	(63.5–88.3)	75.0	(62.0–85.0)	70.0	(63.0–82.0)
Missing values	113	(30.5)	63.0	(28.8)	36.0	(31.6)	13.0	(17.1)	5.0	(8.6)
**BMI**, median (IQR), kg/m2	25.4	(22.6–28.2)	25.5	(22.5–28.8)	24.80	(22.8–28.8)	24.80	(22.2–27.7)	24.50	(21.3–27.1)
Underweight (%)	13	(3.5)	7	(3.2)	4	(3.5)	4	(5.3)	6	(10.3)
Normal weight (%)	104	(28.1)	61	(27.9)	37	(32.5)	29	(38.2)	26	(44.8)
Overweight (%)	93	(25.1)	55	(25.1)	24	(21.1)	21	(27.6)	15	(25.9)
Obesity I°(%)	29	(7.8)	21	(9.6)	8	(7.0)	7	(9.2)	5	(8.6)
Obesity II°(%)	11	(3.0)	8	(3.7)	4	(3.5)	1	(1.3)	1	(1.7)
Obesity III°(%)	4	(1.1)	2	(0.9)	0	(0.0)	0	(0.0)	0	(0.0)
Missing (%)	116	(31.4)	65	(29.7)	37	(32.5)	14	(18.4)	1	(1.7)
**ALBUMIN**, median (IQR), g/l	43.4	(40.2–45.9)	43.5	(40.1–45.8)	44.9	(42.0-46.7)	43.9	(41.9–46.0)	44.0	(42.3–46.4)
Normal	311	(84.1)	181	(82.6)	95	(83.3)	65	(85.5)	49	(84.5)
Low (%)	16	(4.3)	9	(4.1)	3	(2.6)	1	(1.3)	0	(0.0)
Missing (%)	43	(11.6)	29	(13.2)	16	(14.0)	10	(13.2)	9	(15.5)
CRP, median (IQR), mg/l	6.3	(1.0-29.3)	8.7	(2.0-42.7)	2.0	(1.0-12.1)	3.0	(1.0-15.3)	2.0	(1.0-5.8)
Missing (%)	44	(12.0)	20.0	(9.1)	9.0	(7.9)	13.0	(17.1)	0.0	(0.0)
**HEMOGLOBIN**, median (IQR), g/dl	12.9	(11.4–14.2)	12.8	(11.3–14.1)	13.6	(12.2–14.7)	13.6	(12.3–14.4)	13.0	(12.2–14.3)
No anemia (%)	220	(59.5)	126	(57.5)	83	(72.8)	52	(68.4)	36	(62.1)
Mild anemia (%)	79	(21.4)	48	(21.9)	17	(14.9)	10	(13.2)	7	(12.1)
Moderate anemia (%)	69	(18.6)	44	(20.1)	14	(12.3)	14	(18.4)	15	(25.9)
Severe anemia (%)	2	(0.5)	1	(0.5)	0	(0.0)	0	(0.0)	0	(0.0)
Missing (%)	0.0	(0.0)	0.0	(0.0)	0.0	(0.0)	0.0	(0.0)	0.0	(0.0)

*IQR* interquartile range, *BMI* body mass index

**Table 3:** Predictors of overall survival in patients after resection of primary tumors and first to third tumor recurrences, according to univariable (log-rank) and multivariable (COX) analysis

OVERALL Survival																
	**PRIMARY TUMORS**				**1** ^**ST**^ **recurrence**				**2** ^**ND**^ **recurrence**				**3** ^**RD**^ **recurrence**			
	***p*** **-value** **(log-rank)**	**HR**	**95% CI**	***p*** **-value** **(Cox)**	***p*** **-value** **(log-rank)**	**HR**	**95% CI**	***p*** **-value** **(Cox)**	***p*** **-value** **(log-rank)**	**HR**	**95% CI**	***p*** **-value** **(Cox)**	***p*** **-value** **(log-rank)**	**HR**	**95% CI**	***p*** **-value** **(Cox)**
**AGE**	<0.001	1.05	1.03–1.08	**<0.001**	0.060	1.02	1-1.05	0.081	0.073	1.01	0.98–1.03	0.714	0.341			
**ASA SCORE** ASA 3/4 vs. ASA 1/2	0.006	1.44	0.93–2.23	0.103	0.006	2.09	1.13–3.87	**0.019**	0.997				0.596			
**TUMOR HISTOLOGY**	<0.001			**0.001**	0.208				0.745				0.806			
WD-LPS vs. DD-LPS		1.02	0.26–3.95	0.981												
LMS vs. DD-LPS		2.70	1.53–4.76	**0.001**												
NOS vs. DD-LPS		1.95	0.81–4.72	0.137												
Other vs. DD-LPS		4.33	1.92–9.78	**<0.001**												
SFT vs. DD-LPS		0.84	0.1–7.12	0.870												
**TUMOR GRADE**	<0.001			**0.033**												
N.a. vs. G1		2.04	0.55–7.58	0.285												
G2 vs. G1		2.38	0.68–8.33	0.173												
G3 vs. G1		4.16	1.18–14.67	**0.027**												
**TUMORSIZE**	0.446				0.362				0.595				0.530			
**MULTIFOCALITY** yes vs. no	0.003	1.92	0.81–4.56	0.138	0.796				0.767				0.592			
**MVR** yes vs. no	0.002	1.96	1.21–3.17	**0.006**	0.308				0.002				0.669			
**RESECTION STATUS** **R2 vs. R0/R1**	<0.001	1.44	0.86–2.15	0.189	0.003	2.07	1.14–3.77	**0.018**	<0.001	2.42	1.34–4.37	**0.003**	<0.001			
**HEMOGLOBIN**	0.001	0.87	0.77–0.98	**0.020**	0.061	1.17	0.94–1.45	0.172	<0.001	078	0.62–0.97	**0.027**	0.001	0.74	0.55-1.00	**0.050**
**ALBUMIN**	0.278				<0.001	0.89	0.84–0.95	**0.001**	< 0.001	1.06	0.95–1.18	0.318	< 0.001	0.93	0.83–1.05	0.222

*ASA* American Society of Anesthesiologists; *CCI* Comprehensive Complication Index. *DD-LPS* dedifferentiated liposarcoma; *WD-LPS* well-differentiated liposarcoma *LMS* leiomyosarcoma;; *NOS* undifferentiated sarcoma (not otherwise specified); *N.A*. not applicable; *MVR* multivisceral resection. Missing values due to stepwise variable selection in cases of low number of events

Concerning serum albumin levels, only a small proportion of patients exhibited values below the reference range. Concomitant serum CRP levels were low, excluding general inflammation as a potential cause for low serum albumin.

At primary presentation, 150 patients (41%) presented with low Hb values. Of those, 71 patients (19%) were diagnosed with moderate or severe anemia. Similar proportions apply to the subgroup of patients with primary tumors. In the entire cohort as well as all subgroups, the majority of these anemias were normochromic and normocytic. However, a notable proportion was microcytic and/or hypochromic (20–40%), suggesting potential iron deficiency (see Supplementary Table 1).

### Alteration in nutritional parameters upon repeated resection of RPS

**Fig. 2:**
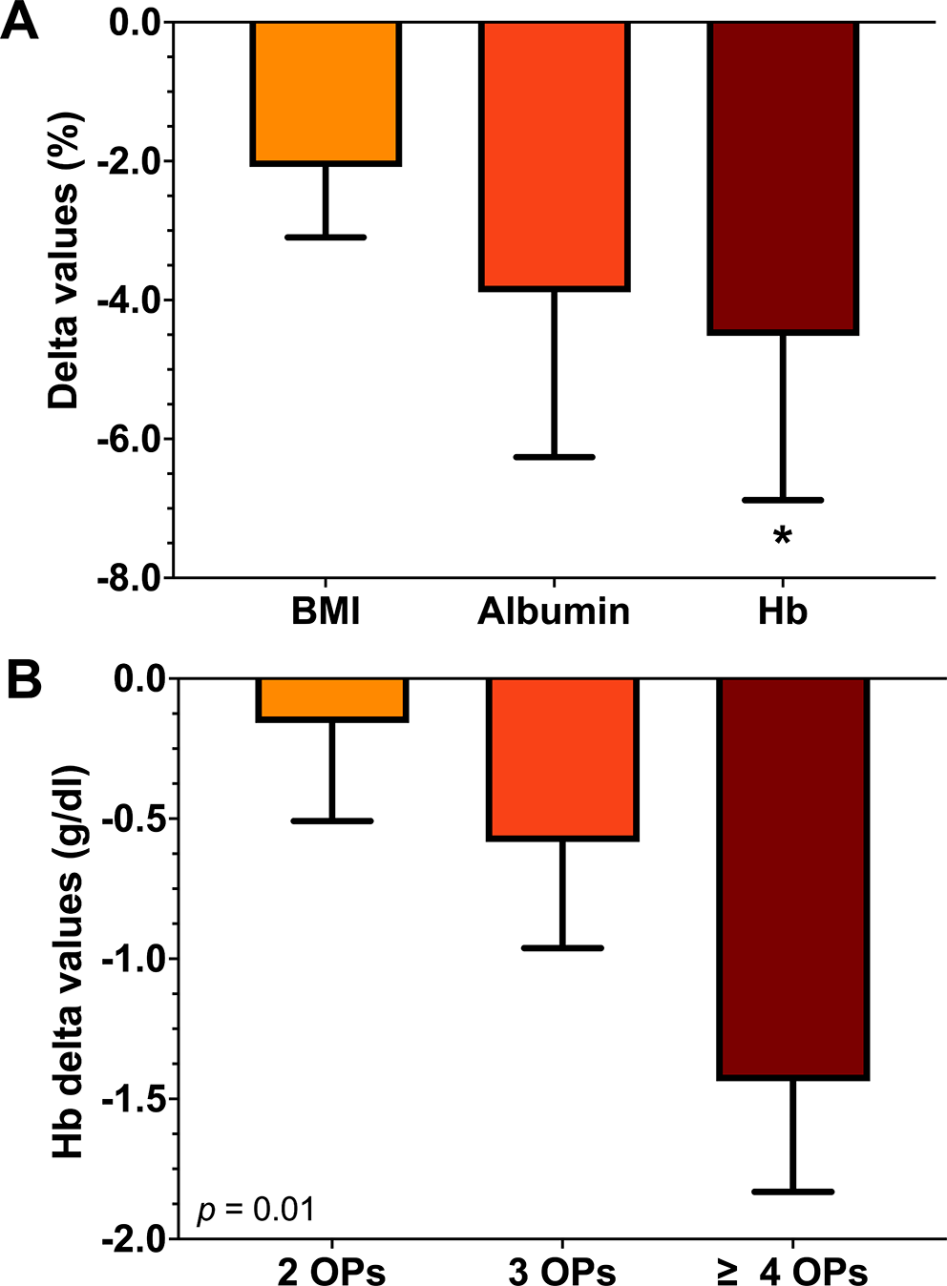
Alterations in nutritional parameters *upon repeated resection of RPS* are illustrated as delta values, calculated as the difference between values before initial and final operations. Negative values indicate a decrease, while positive values indicate an increase over time. The transformation in nutritional parameters (**A**) is presented as delta values as a percentage of the initial value. The variation in hemoglobin values dependent on the number of tumor resections (**B**), is depicted as absolute delta values (g/dl) (**B**). *p < 0.05

In patients undergoing repeated tumor resection, BMI and serum albumin exhibited no significant change over time (all *p* > 0.05). By contrast, hierarchical linear regression analysis (HLM) revealed a significant decrease of Hb values over the course of the disease (β = − 0.98 ± 0.43, *t*= -2.3, *df* = 345, *p* = 0.022, *r* = 0.12) (see Fig. 2a). Intriguingly, the decline in Hb was significantly associated with an increasing number of RPS resections (β = 0.30 ± 0.11, *t* = 2.3, *df* = 365, *p* = 0.010, *r* = 0.13) (see Fig. 2b). However, decreasing Hb values were not associated with the performance of MVR or the cumulative complication burden (all *p* > 0.05).

### Significance of preoperative nutritional and anemia status on morbidity, blood transfusion, and length of hospital stay

Multivariable linear regression models were employed to identify parameters associated with postoperative complications (CCI) and length of hospital stay (LoS) (see Supplementary Tables 2 and 3). In the entire cohort, only higher patient age at the time of the operation was found to be associated with an increased likelihood of postoperative morbidity (β: 0.26 ± 0.11; *p* = 0.017). A similar pattern of findings was observed in the analysis focusing on primary tumors alone (β: 0.25 ± 0.11; *p* = 0.029). Neither low albumin nor low Hb values were linked to the occurrence of postoperative complications. However, 30-day mortality was significantly higher in patients with preoperative anemia compared to patients with normal Hb-values after resection of a primary tumor (9.4% vs. 0.8%; *p* = 0.004). A similar trend was observed for resection of first and second recurrences, but did not reach statistical significance (first: 3.3% vs. 0.0%; *p* = 0.100; second: 14.3% vs. 1.9%; *p* = 0.055).

Concerning LoS, surrogate parameters for nutritional status showed no association with prolonged LoS in either the overall cohort or any subgroup analyses (all *p* > 0.05, see Supplementary Table 3).

Logistic regression models were used to investigate the impact of nutritional parameters on the necessity of perioperative transfusions of blood components (see Supplementary Table 4). As expected, the need for perioperative blood component transfusions within the entire cohort was significantly associated with lower preoperative Hb levels (OR: 0.702; 95% CI: 0.594–0.83; *p* < 0.001) and increased intraoperative blood loss (OR: 1.002; 95% CI. 1.001–1.002; *p* < 0.001). Similar associations were observed concerning the subgroup of primary tumors (Hb: OR: 0.669; 95% CI: 0.527–0.849; *p* = 0.001; blood loss: OR: 1.002; 95% CI: 1.001–1.002; *p* < 0.001). Notably, in the case of tumor recurrences, the transfusion of blood components was independent from preoperative Hb values, and manifested an association solely with intraoperative blood loss (first recurrence: OR: 1.001 95% CI: 1.000-1.002; *p* = 0.001; second recurrence: OR: 1.003; 95% CI: 1.001–1.005; *p* = 0.002). Neither preoperative albumin levels nor the performance of MVR had an impact on perioperative blood transfusions across any of the cohorts.

### Impact of nutritional parameters on overall survival

**Fig. 3:**
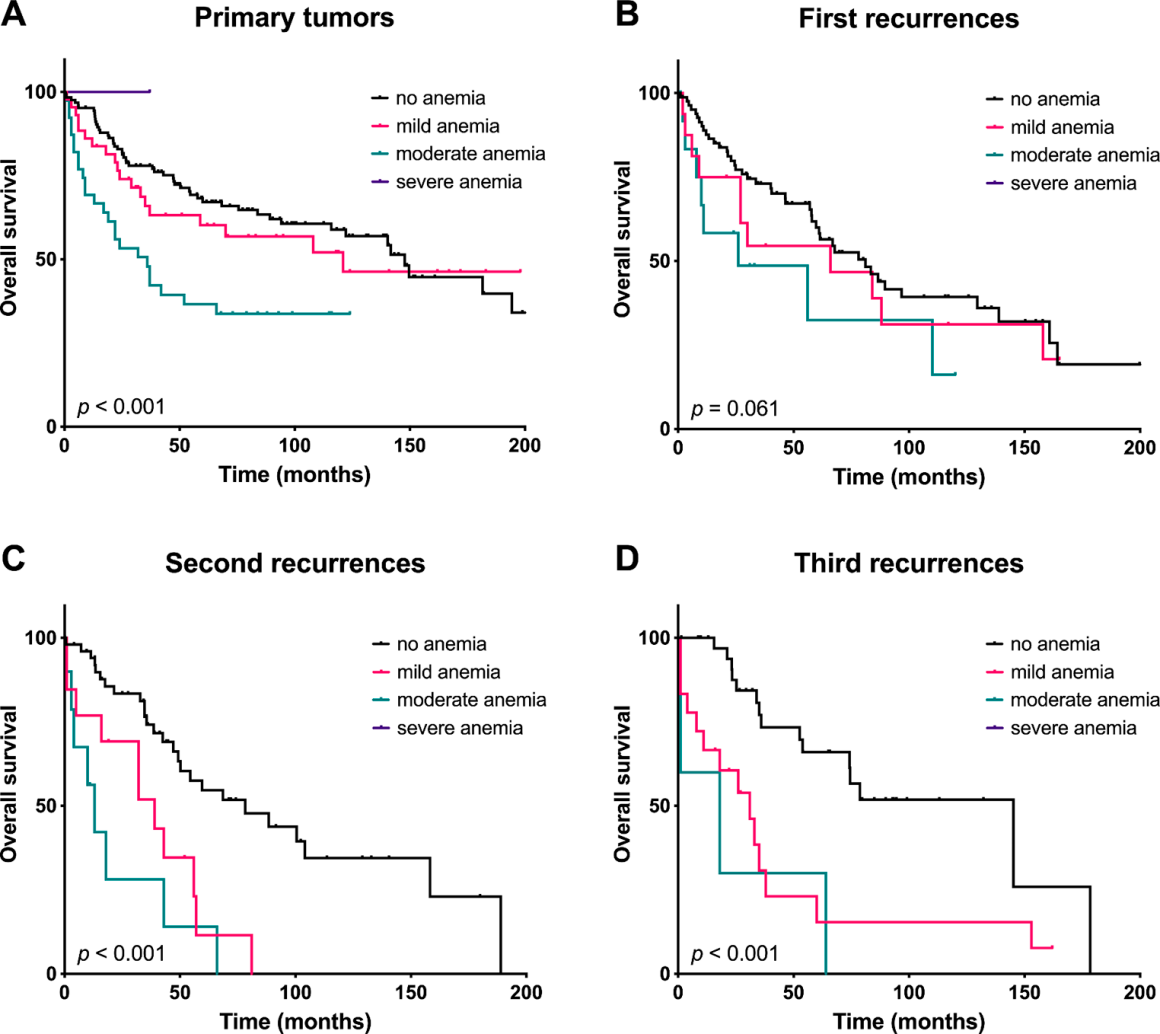
Kaplan-Meier curves with log-rank test, indicating overall survival after resection of primary tumors (**A**) as well as after resection of first (**B**), second (**C**) and third tumor recurrences (**D**), depending on preoperative hemoglobin levels. Classification of anemia according to WHO: mild: Hb 11.0–11.9 g/dl in women; Hb 11.0–12.9 g/dl in men, moderate: Hb 8.0–10.9 g/dl, severe: Hb < 8.0 g/dl

The median overall survival (OS) for the entire cohort following initial presentation at Heidelberg University Hospital was 68.5 months (95% CI: 53.5–83.6). Specifically for primary tumors, the median OS was 121.5 months (95% CI: 74.2–168.8). Multivariable Cox regression analyses were conducted to explore the impact of nutritional parameters after the first to fourth tumor resection (see Table 3). Preoperative serum albumin levels did not exert an influence on OS regarding the resection of primary tumors or the resection of second and third recurrences (all *p* > 0.05). Notably, only following the resection of the first recurrence low preoperative albumin levels were associated with reduced OS (HR = 0.89; 95% CI: 0.84–0.95; *p* = 0.001). Importantly, in this subgroup, serum albumin was below the reference value in only 3 patients (2.6%). Interestingly, low Hb values were independently linked to reduced OS after the resection of primary tumors (HR = 0.87; 95% CI: 0.77–0.98; *p* = 0.020), second recurrences (HR = 0.78; 95% CI: 0.62–0.97; *p* = 0.027), as well as third recurrences (HR = 0.74; 95% CI: 0.55-1.00; *p* = 0.050) (see Fig. 3). However, in patients with reduced Hb-values the etiology of anemia did not affect survival rates (see Supplementary Fig. 1).

## Discussion

To our knowledge, this is the first study to retrospectively evaluate nutritional and anaemic status during the course of RPS, with a focus on multivisceral and repeated tumor resections.

Using albumin and BMI to assess nutritional status, we found no severe malnutrition in our patient cohort, which is in contrast to previous studies where malnutrition rates in RPS patients were as high as 46–52% [13, 14]. Hypoalbuminemia was less common in our cohort (< 5%) compared to other RPS cohorts (16%) [13, 19], which may explain the lack of association with increased morbidity or prolonged hospital stay reported in previous studies [13, 14, 19]. Another reason may be that the assessment of nutritional status in this analysis differed from the aforementioned studies. Due to its retrospective nature, this study evaluated standard preoperative parameters, which may not be ideal for a comprehensive assessment of nutritional status. Furthermore, BMI, although widely used, may not be a reliable reflection of nutritional status in RPS due to the weight of the tumor (up to 20 kg). In addition, obesity may mask malnutrition and catabolic states [15, 20]. Thus, current methods, including albumin or BMI-based scores, may not be sufficient to assess nutritional status in RPS, highlighting the need for RPS-specific tools, particularly in obese patients.

While the majority of patients in our cohort demonstrated normal albumin levels, a notable prevalence of anemia (40%) was observed, contrasting with the 4.3% rate in the general German population over 65 years old [21], as well as the approximately 25% rate in hospitalized patients admitted for surgery aged 50–80 years [22]. Of these anemic patients microcytic and/or hypochromic anemia, suggestive of iron deficiency, was present in 20–40% of patients, consistent with findings by Eisele et al., identifying 29% of anemias in the German population as attributable to iron deficiency [21]. Preoperative Hb values decreased over the disease course, which was independently associated with the number of resections performed but not with postoperative complications or MVR. Importantly, in our cohort, low preoperative Hb levels were identified as an independent prognostic factor for impaired overall survival after resection of primary tumors and recurrences, regardless of the etiology of anemia. Preoperative anemia has previously been associated with higher surgical complication rates, longer hospital stays and increased in-hospital mortality [22]. In our cohort, multiple regression analysis revealed no association between low Hb levels and complication rates or LoS, but higher 30-day mortality. Low preoperative Hb levels reportedly correlate with higher transfusion rates of blood components [22]. However, in our cohort, a correlation between preoperative anemia and the need for blood transfusions was only observed in primary tumors. This observation is consistent with findings by Wong et al., who showed that the need for blood transfusions in RPS patients depends predominantly on the complexity of the surgery and worse tumor biology than on preoperative anemia [23]. Interestingly, in gastric, colon, and bladder cancer, blood component transfusions have been associated with higher tumor recurrence rates [24–26]. Wong et al. did not observe worse overall or disease-free survival in RPS patients who received blood transfusions in multivariable analysis. However, as the authors observed strong associations between the need of transfusion of blood components and oncologic outcomes in univariate analysis, they argue that potential independent effects may be outweighed by tumor biology and comorbidity status [23]. Eventually, potential associations between low Hb values and impaired survival remain unexplained, whether as a reflection of comorbidity status, poor tumor biology, transfusion of blood components, higher in-hospital mortality, or, probably most likely, a combination of these factors. Nevertheless, improvement of Hb levels, especially in case of iron deficiency, which may constitute up to 40%, appears to be a prudent therapeutic approach. Vigilant monitoring of preoperative Hb levels as well as during follow-up is paramount, considering our observation of a substantial decline in Hb values during the course of the disease. This may facilitate prompt diagnosis of anemia, and allows for timely initiation of therapy, potentially contributing to improved patient outcomes and survival.

Major limitations of our present study reside in its retrospective nature, inherently carrying the risk of selection bias and incomplete data collection. As discussed above, we were constrained in the selection of additional parameters for assessing nutritional status, potentially overlooking relevant indicators. Moreover, the absence of comprehensive data on patient comorbidities likewise represents a study limitation. These factors could confound associations between nutritional parameters and outcomes. Despite these limitations, the study has several strengths. To our knowledge, this study represents the largest cohort to date investigating nutritional status in RPS patients. Furthermore, it is the first to explore changes in nutritional status over the course of the disease - a critical consideration given the frequent need for multiple tumor resections in this patient population. Notably, our study likewise is the first to investigate the role of preoperative anemia in RPS patients. In this regard, we observed a considerable prevalence of preoperative anemia, and identified low preoperative Hb values as an independent factor associated with impaired overall survival. Recently it has been demonstrated that nutritional screening and prehabilitation with oral high-protein nutritional support can mitigate malnutrition, leading to improved outcomes [13]. A similar prehabilitational approach should be considered for monitoring and managing Hb levels in RPS patients, with the aim of optimizing patient outcomes and enhancing survival rates.

## Electronic supplementary material

Below is the link to the electronic supplementary material.

Supplementary Material 1

## Data Availability

Research data can be assessed upon request for specific scientific projects.Please contact Franziska.willis@chiru.med.uni-giessen.de.

## Abbreviations

ASA: American Society of Anesthesiologists
BMI: Body mass index
CCI: Comprehensive Complication Index
CI: Confidence interval
CRP: C-reactive protein
DD-LPS: Dedifferentiated liposarcoma
DM: Distant metastases
FNCLLCC: Fédération Nationale Des Centres de Lutte Contre Le Cancer
Hb: Hemoglobin
HLM: Hierarchical linear models
IQR: Interquartile range
LR: Local recurrence
LMS: Leiomyosarcoma
LoS: Length of stay
LPS l: Liposarcoma
MCH: Mean corpuscular hemoglobin
MCV: Mean corpuscular volume
MVR: Multivisceral resection
NOS: Undifferentiated sarcoma (not otherwise specified)
OS: Overall survival
RPS: Retroperitoneal soft tissue sarcoma
SFT: Solitary fibrous tumor & other tumors with intermediate malignancy
WHO: World Health Organization
