# Supplementary material for: Temporal variation in nutritional status and preoperative anemia among patients with retroperitoneal soft tissue sarcoma: a retrospective longitudinal cohort study
Source: Langenbecks Arch Surg. 2025 Jan 22;410(1):48. doi: 10.1007/s00423-024-03585-5 (PMC11754355; doi:10.1007/s00423-024-03585-5)
Supplement: Supplementary file 1 — Supplementary Material 1 [file 423_2024_3585_MOESM1_ESM.docx]

**Supplementary Table 1**

Prevalence and Classification of Anemia Subtypes.

|  | ALL PATIENTS | | PRIMARY TUMORS | | 1^st^ recurrence | | 2^nd^ recurrence | | 3^rd^ Recurrence | |
| --- | --- | --- | --- | --- | --- | --- | --- | --- | --- | --- |
| n | 370 |  | 219 |  | 114 |  | 76 |  | 58 |  |
| No anemia (%) | 220 | (59.5) | 126 | (57.5) | 83 | (72.8) | 52 | (68.4) | 36 | (62.1) |
| Mild anemia (%) | 79 | (21.4) | 48 | (21.9) | 17 | (14.9) | 10 | (13.2) | 7 | (12.1) |
| Moderate anemia (%) | 69 | (18.6) | 44 | (20.1) | 14 | (12.3) | 14 | (18.4) | 15 | (25.9) |
| Severe anemia (%) | 2 | (0.5) | 1 | (0.5) | 0 | (0.0) | 0 | (0.0) | 0 | (0.0) |
| Microcytic (%) | 6 | (1.6) | 5 | (2.3) | 0 | (0.0) | 1 | (1.3) | 0 | (0.0) |
| Normocytic (%) | 6 | (1.6) | 3 | (1.4) | 1 | (0.9) | 0 | (0.0) | 0 | (0.0) |
| Hypochromic, microcytic (%) | 0 | (0.0) | 12 | (5.5) | 4 | (3.5) | 3 | (3.9) | 1 | (1.7) |
| Hypochromic, normocytic (%) | 21 | (5.7) | 18 | (8.2) | 1 | (0.9) | 1 | (1.3) | 4 | (6.9) |
| Normochromic, microcytic (%) | 23 | (6.2) | 0 | (0.0) | 24 | (21.1) | 1 | (1.3) | 1 | (1.7) |
| Normochromic, normocytic (%) | 1 | (0.3) | 49 | (22.4) | 24 | (211) | 15 | (19.7) | 15 | (25.9) |
| Normochromic, macrocytic (%) | 83 | (22.4) | 4 | (1.8) | 1 | (09) | 3 | (3.9) | 1 | (1.7) |
| Hyperchromic, normocytic (%) | 8 | (2.2) | 0 | (0.0) | 0 | (00) | 0 | (0.0) | 0 | (0.0) |
| Hyperchromic, macrocytic (%) | 0 | (0.0) | 2 | (0.9) | 0 | (0.0) | 0 | (0.0) | 0 | (0.0) |

**Supplementary Table 2**

Association between postoperative complications as quantified by the Comprehensive Complication Index (CCI), and clinical/demographic variables according to multivariable linear regression models. Regression models were only calculated when the ANOVA indicated a good fit for the data (*p* ≤ 0.05).

| CCI | Entire cohort |  |  |  |  |
| --- | --- | --- | --- | --- | --- |
| ANOVA | **R^2^** | ***df*** | **F** | ***f^2^*** | ***p*-VALUE** |
|  | 0.069 | 6; 280 | 3.456 | 0.074 | **0.003** |
| REGRESSION |  | **Parameter estimate (mean ± SE)** | **95 % CI** | | ***p*-VALUE** |
| Age |  | 0.26 ± 0.11 | 0.05-0.46 | | **0.017** |
| Hemoglobin |  | -1.04 ± 0.97 | -2.94-0.83 | | 0.300 |
| ALbumin |  | -0.05 ± 0.39 | -0.79-0.74 | | 0.914 |
| MVR | yes vs. no | 5.71 ± 2.99 | -0.22-11.30 | | 0.061 |
| Tumorsize |  | -0.06 ± 0.12 | -0.28-0.18 | | 0.655 |
| ASA Score | 3/4 vs. 1/2 | 4.83 ± 2.86 | -0.66-10.72 | | 0.096 |
| CCI | **Primary Tumors** | |  |  |  |
| ANOVA | **R^2^** | ***df*** | **F** | ***f^2^*** | ***p*-VALUE** |
|  | 0.081 | 6; 163 | 2.406 | 0.089 | **0.030** |
| REGRESSION |  | **Parameter estimate (mean ± SE)** | **95 % CI** | | ***p*-VALUE** |
| Age |  | 0.25 ± 0.11 | 0.02-0.45 | | **0.029** |
| Hemoglobin |  | -2.31 ± 1.28 | -4.79-0.21 | | 0.074 |
| ALbumin |  | 0.37 ± 0.41 | -0.39-1.11 | | 0.417 |
| MVR | yes vs. no | 2.31 ± 3.73 | -5.05-9.51 | | 0.566 |
| Tumorsize |  | -0.1 ± 0.13 | -0.35-0.17 | | 0.486 |
| ASA Score | 3/4 vs. 1/2 | 4.68 ± 3.96 | -3.02-12.59 | | 0.255 |
| CCI | **First REcurrences** | |  |  |  |
| ANOVA | **R^2^** | ***df*** | **F** | ***f^2^*** | ***p*-VALUE** |
|  | 0.120 | 6; 80 | 1.826 | 0.137 | 0.104 |
| CCI | **Second REcurrences** | |  |  |  |
| ANOVA | **R^2^** | ***df*** | **F** | ***f^2^*** | ***p*-VALUE** |
|  | 0.155 | 6; 52 | 1.595 | 0.184 | 0.167 |
| CCI | **Third REcurrences** | |  |  |  |
| ANOVA | **R^2^** | ***df*** | **F** | ***f^2^*** | ***p*-VALUE** |
|  | 0.314 | 6; 36 | 2.749 | 0.458 | **0.026** |
| REGRESSION |  | **Parameter estimate (mean ± SE)** | **95 % CI** | | ***p*-VALUE** |
| Age |  | 0.18 ± 0.15 | -0.08-0.50 | | 0.241 |
| Hemoglobin |  | 1.52 ± 1.44 | -1.02-4.45 | | 0.341 |
| ALbumin |  | -0.85 ± 0.84 | -2.57-0.77 | | 0.327 |
| MVR | yes vs. no | 18.65 ± 9.86 | -2.83-35.22 | | 0.107 |
| Tumorsize |  | 0.50 ± 0.33 | -0.19-1.10 | | 0.165 |
| ASA Score | 3/4 vs. 1/2 | 0.28 ± 5.40 | -10.93-10.10 | | 0.961 |

*ASA* American Society of Anesthesiologists, *CI* confidence interval, *CCI* Comprehensive Complication Index, *LOS* length of hospital stay*, SE* standard deviation.

**Supplementary Table 3**

Association between the length of hospital stay (LOS) and clinical/demographic variables according to multivariable linear regression models. Regression models were only calculated when the ANOVA indicated a good fit for the data (*p* ≤ 0.05).

| LOS | Entire cohort |  |  |  |  |
| --- | --- | --- | --- | --- | --- |
| ANOVA | **R^2^** | ***df*** | **F** | ***f^2^*** | ***p*-VALUE** |
|  | 0.349 | 7; 279 | 21.406 | 0.537 | **<0.001** |
| REGRESSION |  | **Parameter estimate (mean ± SE)** | **95 % CI** | | ***p*-VALUE** |
| Age |  | -0.02 ± 0.05 | -0.12-0.08 | | 0.671 |
| CCI |  | 0.36 ± 0.07 | 0.21-0.5 | | **<0.001** |
| Hemoglobin |  | -0.53 ± 0.43 | -1.37-0.32 | | 0.228 |
| ALbumin |  | 0.03 ± 0.17 | -0.3-0.36 | | 0.874 |
| MVR | yes vs. no | 4.42 ± 1.7 | 1.32-7.9 | | **0.012** |
| Tumorsize |  | 0.05 ± 0.06 | -0.07-0.17 | | 0.423 |
| ASA Score | 3/4 vs. 1/2 | 3.58 ± 1.7 | 0.07-6.71 | | **0.040** |
| LOS | **Primary Tumors** | |  |  |  |
| ANOVA | **R^2^** | ***df*** | **F** | ***f^2^*** | ***p*-VALUE** |
|  | 0.267 | 7; 162 | 8.443 | 0.365 | **<0.001** |
| REGRESSION |  | **Parameter estimate (mean ± SE)** | **95 % CI** | | ***p*-VALUE** |
| Age |  | -0.06 ± 0.05 | -0.17-0.04 | | 0.273 |
| CCI |  | 0.24 ± 0.09 | 0.07-0.41 | | **0.009** |
| Hemoglobin |  | -0.68 ± 0.5 | -1.66-0.3 | | 0.189 |
| ALbumin |  | -0.15 ± 0.2 | -0.54-0.24 | | 0.478 |
| MVR | yes vs. no | 6.63 ± 1.98 | 2.64-10.3 | | **0.001** |
| Tumorsize |  | 0.02 ± 0.08 | -0.12-0.16 | | 0.892 |
| ASA Score | 3/4 vs. 1/2 | 3.78 ± 2.21 | -0.42-8.17 | | 0.103 |
| LOS | **First REcurrences** | |  |  |  |
| ANOVA | **R^2^** | ***df*** | **F** | ***f^2^*** | ***p*-VALUE** |
|  | 0.518 | 7; 79 | 12.138 | 1.076 | **<0.001** |
| REGRESSION |  | **Parameter estimate (mean ± SE)** | **95 % CI** | | ***p*-VALUE** |
| Age |  | 0.15 ± 0.08 | -0.02-0.31 | | 0.095 |
| CCI |  | 0.67 ± 0.14 | 0.41-0.93 | | **<0.001** |
| Hemoglobin |  | -0.36 ± 0.71 | -1.73-1.01 | | 0.654 |
| ALbumin |  | 0.12 ± 0.33 | -0.49-0.72 | | 0.776 |
| MVR | yes vs. no | -4.89 ± 4.31 | -13.17-3.42 | | 0.300 |
| Tumorsize |  | 0.25 ± 0.23 | -0.16-0.66 | | 0.347 |
| ASA Score | 3/4 vs. 1/2 | -3.12 ± 3.06 | -8.96-2.97 | | 0.346 |
| LOS | **Second REcurrences** | |  |  |  |
| ANOVA | **R^2^** | ***df*** | **F** | ***f^2^*** | ***p*-VALUE** |
|  | 0.543 | 7; 51 | 8.646 | 1.187 | **<0.001** |
| REGRESSION |  | **Parameter estimate (mean ± SE)** | **95 % CI** | | ***p*-VALUE** |
| Age |  | -0.06 ± 0.08 | -0.22-0.10 | | 0.490 |
| CCI |  | 0.25 ± 0.05 | 0.16-0.35 | | **<0.001** |
| Hemoglobin |  | -0.19 ± 0.88 | -1.70-1.31 | | 0.909 |
| ALbumin |  | 0.21 ± 0.20 | -0.17-0.61 | | 0.370 |
| MVR | yes vs. no | 9.97 ± 3.40 | 3.05-16.53 | | **0.011** |
| Tumorsize |  | 0.35 ± 0.16 | 0.04-0.64 | | **0.046** |
| ASA Score | 3/4 vs. 1/2 | 0.11 ± 1.70 | -3.24-3.38 | | 0.955 |
| LOS | **Third REcurrences** | |  |  |  |
| ANOVA | **R^2^** | ***df*** | **F** | ***f^2^*** | ***p*-VALUE** |
|  | 0.791 | 7; 35 | 18.875 | 3.776 | **<0.001** |
| REGRESSION |  | **Parameter estimate (mean ± SE)** | **95 % CI** | | ***p*-VALUE** |
| Age |  | 0.05 ± 0.1 | -0.12-0.26 | | 0.671 |
| CCI |  | 0.71 ± 0.13 | 0.43-0.92 | | **<0.001** |
| Hemoglobin |  | -0.34 ± 0.81 | -1.76-1.36 | | 0.698 |
| ALbumin |  | -0.15 ± 0.45 | -1.1-0.66 | | 0.757 |
| MVR | yes vs. no | 1.77 ± 4.33 | -7.67-8.88 | | 0.751 |
| Tumorsize |  | -0.04 ± 0.14 | -0.34-0.2 | | 0.763 |
| ASA Score | 3/4 vs. 1/2 | 2.57 ± 2.7 | -3.2-7.16 | | 0.387 |

*ASA* American Society of Anesthesiologists, *CI* confidence interval, *CCI* Comprehensive Complication Index, *LOS* length of hospital stay*, SE* standard deviation.

**Supplementary Table 4**

Association between the necessity of blood component transfusion and clinical variables according to logistic regression.

| Blood component Transfusion | Entire cohort |  |  |  |  |
| --- | --- | --- | --- | --- | --- |
| REGRESSION |  | **odds ratio** | **95 % CI** | | ***p*-VALUE** |
| Hemoglobin |  | 0.70 | 0.59-0.83 | | <0.001 |
| ALbumin |  | 1.01 | 0.96-1.07 | | 0.622 |
| MVR | yes vs. no | 1.20 | 0.65-2.22 | | 0.558 |
| Blood loss |  | 1.002 | 1.001-1.002 | | <0.001 |
| Blood component transfusion | **Primary Tumors** | |  |  |  |
| REGRESSION |  | **odds ratio** | **95 % CI** | | ***p*-VALUE** |
| Hemoglobin |  | 0.67 | 0.53-0.85 | | 0.001 |
| ALbumin |  | 1.02 | 0.96-1.09 | | 0.527 |
| MVR | yes vs. no | 0.76 | 0.33-1.76 | | 0.528 |
| Blood loss |  | 1.002 | 1.001-1.002 | | <0.001 |
| blood component transfusion | **First REcurrences** | |  |  |  |
| REGRESSION |  | **odds ratio** | **95 % CI** | | ***p*-VALUE** |
| Hemoglobin |  | 0.87 | 0.63-1.20 | | 0.396 |
| ALbumin |  | 0.97 | 0.85-1.11 | | 0.680 |
| MVR | yes vs. no | 3.80 | 1.14-12.67 | | 0.030 |
| Blood loss |  | 1.001 | 1.000-1.002 | | 0.001 |
| BLOOD Component Transfusion | **Second REcurrences** | |  |  |  |
| REGRESSION |  | **odds ratio** | **95 % CI** | | ***p*-VALUE** |
| Hemoglobin |  | 0.64 | 0.38-1.09 | | 0.102 |
| ALbumin |  | 0.84 | 0.64-1.11 | | 0.221 |
| MVR | yes vs. no | 3.17 | 0.31-32.24 | | 0.329 |
| Blood loss |  | 1.003 | 1.001-1.005 | | 0.002 |
| Blood component transfusion | **Third REcurrences** | |  |  |  |
| REGRESSION |  | **odds ratio** | **95 % CI** | | ***p*-VALUE** |
| Hemoglobin |  | 604.99 | 0-0 | | 0.997 |
| ALbumin |  | 0.04 | 0-0 | | 0.999 |
| MVR | yes vs. no | 0.00 | 0-0 | | 0.995 |
| Blood loss |  | 1.15 | 0-0 | | 0.992 |

*CI* confidence interval, *SE* standard deviation.

**Supplementary Figure 1:** Kaplan-Meier curves with log-rank test, indicating overall survival in patients with preoperative anemia after resection of primary tumors (A), as well as after resection of first (B), second (C) and third tumor recurrences (D), stratified by anemia etiology.
